# Crystal structure of di­aqua­[5,10,15,20-tetra­kis­(4-meth­oxy­phen­yl)porphyrinato-κ^4^ *N*]iron(III) di­aqua­(18-crown-6)potassium bis­(tri­fluoro­methane­sulfonate)–18-crown-6 (1/2)

**DOI:** 10.1107/S2056989015021039

**Published:** 2015-11-11

**Authors:** Leila Ben Haj Hassen, Zouhour Denden, Yoann Rousselin, Habib Nasri

**Affiliations:** a University of Monastir, Laboratoire de Physico-chimie des Matriaux, Faculté des Sciences de Monastir, Avenue de l’environnement, 5019 Monastir, Tunisia; b University of Burgundy, ICMUB–UMR 6302, 9 avenue Alain Savary, 21000 Dijon, France

**Keywords:** crystal structure, iron(III) complex salt, tri­fluoro­methane­sulfonate, porphyrin

## Abstract

In the title compound, [Fe^III^(C_48_H_36_N_4_O_2_)(H_2_O)_2_][K(C_12_H_24_O_6_)(H_2_O)_2_](SO_3_CF_3_)_2_·2C_12_H_24_O_6_, the Fe^III^ atom is situated on an inversion centre and is octa­hedrally coordin­ated by four pyrrole N atoms of the deprotenated 5,10,15,20-tetra­kis­(4-meth­oxy­phen­yl)porphyrinate ligand and two water mol­ecules. The average equatorial Fe—N(pyrrole) bond length [2.043 (6) Å] is consistent with a high-spin (*S* = 5/2) iron(III) metalloporphyrin derivative. The K^+^ cation, which also lies on an inversion centre, is chelated by the six O atoms of one 18-crown-6 mol­ecule and is additionally coordinated by two water mol­ecules in a distorted hexa­gonal–bipyramidal geometry. In the crystal, the cations, anions and one non-coordinating 18-crown-6 mol­ecule are linked by classical O—H⋯O hydrogen bonds and non-conventional C—H⋯O hydrogen bonds, leading to a one-dimensional supra­molecular architecture along [10-1]. The crystal packing is further stabilized by weak C—H⋯π inter­actions involving pyrrole and phenyl rings of the porphyrins, as well as weak C—H⋯F contacts involving the (SO_3_CF_3_)^−^ counter-ion and the 18-crown-6 mol­ecules.

## Related literature   

For the synthesis, see: Gismelseed *et al.* (1990 ▸). For related structures, see: Gismelseed *et al.* (1990 ▸); Denden *et al.* (2015 ▸); Scheidt *et al.* (1979 ▸, 1981 ▸); Cheng *et al.* (1994 ▸); Xu *et al.* (2011 ▸); Ben Haj Hassen *et al.* (2014 ▸).
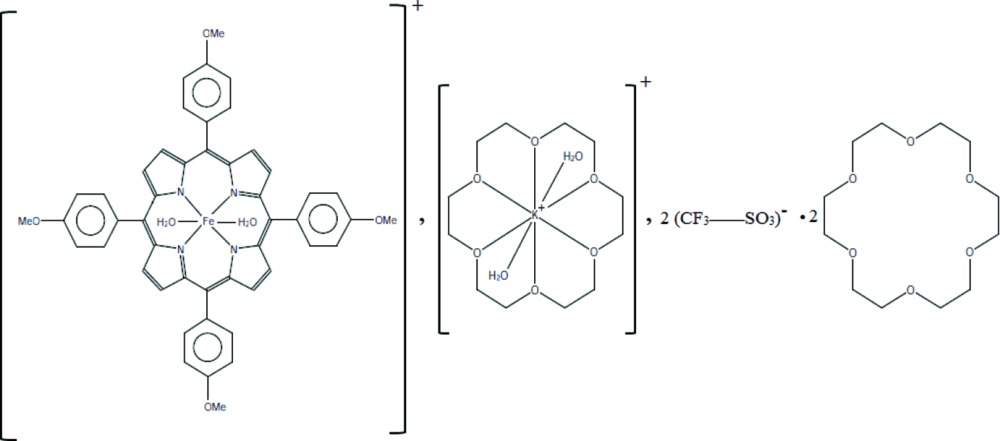


## Experimental   

### Crystal data  

[Fe(C_48_H_36_N_4_O_4_)(H_2_O)_2_][K(C_12_H_24_O_6_)(H_2_O)_2_](CF_3_SO_3_)_2_·2C_12_H_24_O_6_
*M* *_r_* = 1990.89
Triclinic, 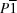

*a* = 12.1294 (4) Å
*b* = 14.1636 (6) Å
*c* = 15.5934 (5) Å
α = 84.081 (3)°
β = 72.567 (2)°
γ = 64.895 (2)°
*V* = 2313.50 (15) Å^3^
*Z* = 1
Mo *K*α radiation
μ = 0.35 mm^−1^
*T* = 115 K
0.52 × 0.50 × 0.50 mm

### Data collection  

Nonius Kappa APEXII diffractometer
Absorption correction: multi-scan (*SADABS*; Bruker, 2012 ▸) *T*_min_ = 0.685, *T*_max_ = 0.746
63523 measured reflections
10707 independent reflections
8615 reflections with *I* > 2σ(*I*)
*R*_int_ = 0.033

### Refinement  

*R*[*F* ^2^ > 2σ(*F* ^2^)] = 0.050
*wR*(*F* ^2^) = 0.146
*S* = 1.07
10700 reflections
602 parameters
H-atom parameters constrained
Δρ_max_ = 0.57 e Å^−3^
Δρ_min_ = −1.75 e Å^−3^

### 

Data collection: *APEX2* (Bruker, 2013 ▸); cell refinement: *SAINT* (Bruker, 2013 ▸); data reduction: *SAINT*; program(s) used to solve structure: *olex2.solve* (Bourhis *et al.*, 2015 ▸); program(s) used to refine structure: *SHELXL97* (Sheldrick, 2008 ▸); molecular graphics: *OLEX2* (Dolomanov *et al.*, 2009 ▸); software used to prepare material for publication: *OLEX2*.

## Supplementary Material

Crystal structure: contains datablock(s) I, 2R. DOI: 10.1107/S2056989015021039/im2471sup1.cif

Structure factors: contains datablock(s) I. DOI: 10.1107/S2056989015021039/im2471Isup2.hkl

**Fig. 1.**
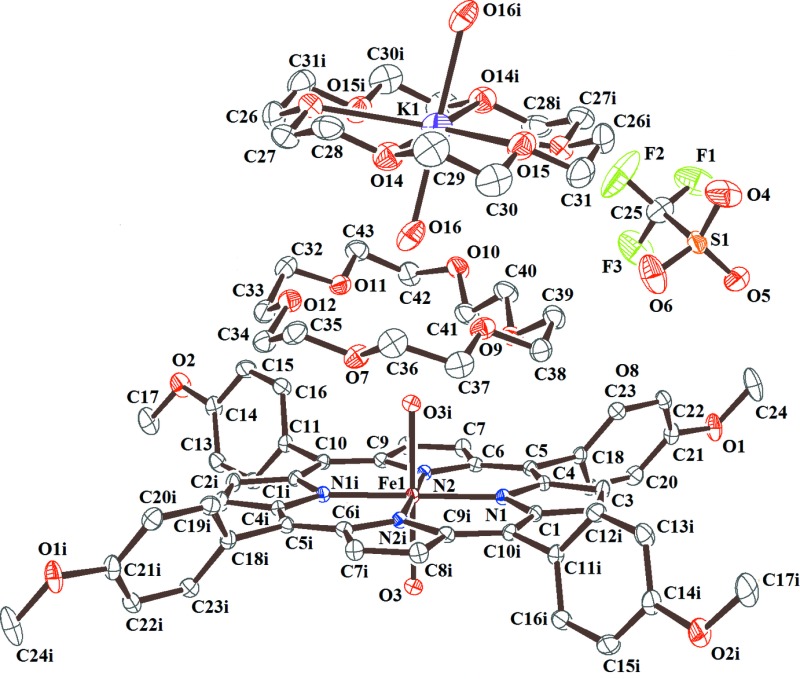
An ORTEP view of the [FeIII(TMPP)(H2O)2]+ and [K(18-crown-6)(H2O)2]+ cations, the (SO3CF3)- anion and the 18-crown-6 molecule. Displacement ellipsoids are drawn at 50% probability level. H atoms have been omitted for clarity. [Symmetry code: (i) 1 + x, y, z].

**Fig. 2.**
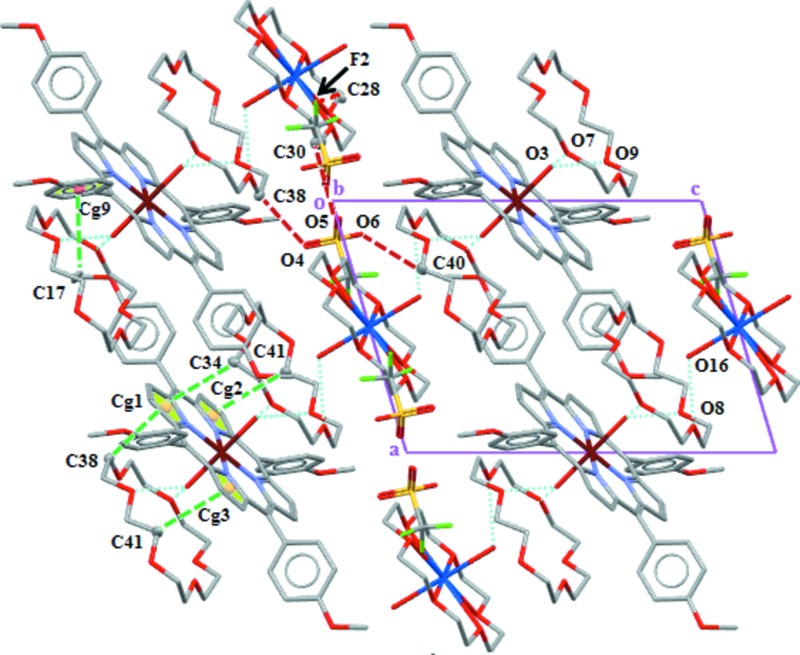
A drawing showing the one-dimensional supramolecular structure of the title compound viewed down the b axis. The O—H···O classic H bonds are drawn as dashed light blue lines, the C—H···O contacts as dashed dark red lines while the C—H···Cg intermolecular interactions are shown as dashed green lines.

ORTEP III 2 2 + 2 2 + 3 3 − x y z . DOI: 10.1107/S2056989015021039/im2471fig1.tif
An *ORTEP* view of the [Fe^III^(TMPP)(H_2_O)_2_]^+^ and [K(18-crown-6)(H_2_O)_2_]^+^ cations, the (SO_3_CF_3_)^−^ anion and the 18-crown-6 mol­ecule. Displacement ellipsoids are drawn at 50% probability level. H atoms have been omitted for clarity. [Symmetry code: (i) 1 + *x*, *y*, *z*].

b Cg . DOI: 10.1107/S2056989015021039/im2471fig2.tif
A drawing showing the one-dimensional supra­molecular structure of the title compound viewed down the *b* axis. The O—H⋯O classic H bonds are drawn as dashed light blue lines, the C—H⋯O contacts as dashed dark red lines while the C—H⋯*Cg* inter­molecular inter­actions are shown as dashed green lines.

CCDC reference: 976053

Additional supporting information:  crystallographic information; 3D view; checkCIF report

## Figures and Tables

**Table 1:** Hydrogen-bond geometry (Å, °) *Cg*1, *Cg*2, *Cg*3 and *Cg*9 ae the centroids of the N1/C1–C4, N2/C6′–C9′, N2′/C6–C9 and C18–C23 rings, respectively.

*D*—H⋯*A*	*D*—H	H⋯*A*	*D*⋯*A*	*D*—H⋯*A*
O3—H3*B*⋯O9^i^	0.87	1.96	2.797 (2)	160
O3—H3*A*⋯O7^i^	0.87	1.94	2.778 (2)	161
O16—H16*A*⋯O8	0.87	2.15	2.922 (3)	147
O16—H16*B*⋯O12	0.87	2.38	3.107 (3)	141
C7—H7⋯O2^ii^	0.95	2.52	3.303 (3)	139
C12—H12⋯O4^iii^	0.95	2.48	3.270 (3)	141
C16—H16⋯O11^iii^	0.95	2.39	3.319 (3)	164
C22—H22⋯O5^iv^	0.95	2.46	3.390 (3)	168
C24—H24*A*⋯O5^v^	0.98	2.46	3.184 (4)	130
C28—H28*A*⋯F2^iii^	0.99	2.50	2.983 (4)	110
C30—H30*B*⋯O5^vi^	0.99	2.49	3.248 (5)	133
C38—H38*A*⋯O4^vi^	0.99	2.55	3.429 (4)	148
C40—H40*B*⋯O6^vii^	0.99	2.51	3.402 (4)	150
C41—H41*A*⋯O11	0.99	2.33	3.030 (4)	127
C17—H17*C*⋯*Cg*9^ii^	0.98	2.84	3.753 (4)	155
C34—H34*B*⋯*Cg*1^viii^	0.99	2.93	3.894 (3)	164
C38—H38*B*⋯*Cg*1^iii^	0.99	2.78	3.706 (3)	155
C41—H41*B*⋯*Cg*2^viii^	0.99	2.95	3.584 (3)	123
C41—H41*B*⋯*Cg*3^iii^	0.99	2.95	3.584 (3)	123

Symmetry codes: (i) 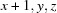; (ii) 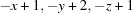; (iii) 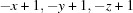; (iv) 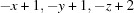; (v) 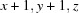; (vi) 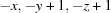; (vii) 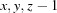; (viii) 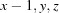.

## supplementary crystallographic information

### S1. Synthesis and crystallization

To a solution of [Fe^III^(TMPP)(SO_3_CF_3_)](Gismelseed *et al.*, 1990) (20 mg, 0.021 mmol) in di­chloro­methane (10 mL) was added an excess of 18-crown-6 (100 mg, 0.378 mmol) and potassium nitrite (60 mg, 0.705 mmol). The reaction mixture was stirred at room temperature and at the end of the reaction, the color of the solution changed from brown red to blood red. Crystals of the title complex were obtained as impurities by diffusion of hexanes through the di­chloro­methane solution. The X-ray analysis was recorded in the "Pôle de Chimie Moléculaire", the technological platform for chemical analysis and molecular synthesis (http://www.wpcm.fr) which relies on the Institute of the Molecular Chemistry of University of Burgundy and Welience "TM", a Burgundy University private subsidiar.

### S2. Comment

In continuation of our research on the crystal structures of porphyrin complexes (Denden *et al.*, 2015) we herein report the synthesis and crystal structure of the title compound.

The asymmetric unit of (I) is made by one half [Fe^III^(TMPP)(H_2_O)_2_]^+^ (TMPP = 5, 10, 15, 20-tetra­kis(4-meth­oxy­phenyl)­porphyrinato) and one half [K(18-crown-6)(H_2_O)_2_]^+^ (18-crown-6 is a crown ether with the formula C_12_H_24_O_6_) cationic complexes, one (SO_3_CF_3_)^-^ counterion and one non-coordinated 18-crown-6 molecule.

It has been noticed for iron(III) porphyrins that there is a relationship between the spin-state of the iron(III) and the value of the average equatorial iron-pyrrole N atoms distance (Fe—Np) (Scheidt & Reed, 1981; Cheng *et al.*, 1994). On the other hand, Fe(III)-mono­aqua porphyrins are inter­mediate-spin (S = 3/2) with an Fe—Np distances around 1.978 Å while Fe(III)-di­aqua metalloporphyrins are high-spin (S = 5/2) with an Fe—Np distance around 2.045 Å (Cheng *et al.*, 1994) . Thus, for [Fe^III^(TPP)(H_2_O)]^+^ (Xu *et al.*, 2011), the Fe—Np distance is 1.982 (3) Å and [Fe^III^(TClPP)(H_2_O)_2_]^+^ (TClPP is the 5, 10, 15, 20-tetra­(para-chloro­phenyl)­porphyrinato ligand) exhibits an Fe—Np bond length of 2.042 (2) Å (Ben Haj Hassen *et al.*, 2014). For Fe(III) mixed-ligands porphyrins type [Fe^III^(Porph)(H_2_O)(L)]^+^ (Porph = porphyrinato) the spin state depends on the nature of the axial L ligand. For example, the Fe—Np distance is 2.022 (8) Å for [Fe^III^(TpivPP)(SO_3_CF_3_)(H_2_O)] (TpivPP is the α,α,α,α-tetra­kis(*o*-pivalamido­phenyl)­porphinato ligand) leading to a mixed inter­mediate-spin derivative [S = (5/2,3/2)] (Gismelseed *et al.*, 1990). The Fe—Np bond length value of our derivative [Fe^III^(TMPP)(H_2_O)_2_]^+^ which is 2.0428 (16) Å is an indication that this species is high-spin (S = 5/2). For (I), the axial Fe—O(H_2_O) bond length is 2.1048 (14) Å while for the [Fe^III^(TPP)(H_2_O)_2_]^+^ and [Fe^III^(ClTPP)(H_2_O)_2_]^+^ related species (Scheidt *et al.*, 1979; Ben Haj Hassen, 2014), the values of this distance are 2.095 (2) Å and 2.051 (2) Å / 2.157 (2) Å, respectively . These bond lengths are comparable to those of several iron(III)-aqua porphyrin complexes [1.961 (3) Å - 2.134 (6) Å] (CSD refcodes ECADET; Xu *et al.*, 2011 and SICFAL; Gismelseed *et al.*, 1990) (CDS, version 5.35). The o­cta-coordinated K^+^ ion is bonded to the six O atoms of the 18-crown-6 molecule with an average K–O(18-crown-6) bond length of 2.780 (2) Å and to the oxygen atoms of the two trans aqua axial ligands with a K—O(H_2_O) bond length of 2.484 (2) Å.

The crystal structure of the title compound resembles to an one-dimensional coordination polymer (Fig. 2). Indeed, each porphyrin [Fe^III^(TMPP)(H_2_O)_2_]^+^ cation is linked via strong O—H···O H bonds to two symmetry-related 18-crown-6 molecules through the O3 and O3i atoms (symmetry code: (i) 1+x, y, z) of the aqua axial ligands of the iron moiety and the oxygens O7/O9 (and O7'/O9') of the two trans 18-crown-6 molecules (O3—H3A···O7 and O3—H3B···O9 distances are 2.778 (2) Å and 2.797 (2) Å, respectively). On the other hand, the symmetry related 18-crown-6 molecule is also hydrogen bonded to the oxygen O16 of the aqua axial ligands of the [K(18-crown-6)(H_2_O)_2_]^+^ cation through the oxygen O8 (O16—H16···O8 bond length is 2.922 (3) Å). This leads to a one-dimensional supra­molecular network which is further consolidated by weak C—H···π inter­actions involving the centroids Cg1, Cg2, Cg3 and Cg9 of pyrroles and phenyls rings of the porphyrins and carbons of adjacent 18-crown-6 molecules (Fig. 2, Table 1).

Notably, the oxygen atoms O4, O6 and O5 of the (SO_3_CF_3_)^-^ counterion are weakly linked to the carbon C38, C40 and C30 of two adjacent 18-crown-6 molecules and the [K(18-crown-6)(H_2_O)_2_]^+^ cation respectively (C38—H38···O4, C40—H40B···O6 and C30—H30B···O5 distances are 3.492 (4) Å, 3.402 (4) Å and 3.248 (5) Å respectively). The fluorine F2 of the same species is weakly bonded via C—H···F contacts to carbon C28 of a neighboring 18-crown-6 molecule (C28—H28A···F2 bond length is 2.983 (4) Å).

### S2.1. Refinement

The positions of H atoms of the two aqua ligands coordinated to Fe(III) and the two aqua ligands coordinated to the potassium of the [K(18-crown-6)(H_2_O)_2_]^+^ counterion, were found in difference maps and then refined with U_iso_(H) = 1.5U_eqiso_(H) = 1.2U_eq_(C_methyl­ene_, _methyl_, _aromatic_).

### Crystal data

**Table:**

[Fe(C_48_H_36_N_4_O_4_)(H_2_O)_2_][K(C_12_H_24_O_6_)(H_2_O)_2_](CF_3_SO_3_)_2_·2C_12_H_24_O_6_	*Z* = 1
*M**_r_* = 1990.89	*F*(000) = 1047
Triclinic, *P*1	*D*_x_ = 1.429 Mg m^−^^3^
*a* = 12.1294 (4) Å	Mo *K*α radiation, λ = 0.71073 Å
*b* = 14.1636 (6) Å	Cell parameters from 9914 reflections
*c* = 15.5934 (5) Å	θ = 2.6–27.6°
α = 84.081 (3)°	µ = 0.35 mm^−^^1^
β = 72.567 (2)°	*T* = 115 K
γ = 64.895 (2)°	Cube, dark blue
*V* = 2313.50 (15) Å^3^	0.52 × 0.50 × 0.50 mm

### Data collection

**Table:**

Nonius Kappa APEXII diffractometer	10707 independent reflections
Radiation source: X-ray tube, Siemens KFF Mo 2K-180	8615 reflections with *I* > 2σ(*I*)
Graphite monochromator	*R*_int_ = 0.033
Detector resolution: 512 x 512 pixels mm^-1^	θ_max_ = 27.6°, θ_min_ = 1.4°
φ and ω scans	*h* = −15→15
Absorption correction: multi-scan (*SADABS*; Bruker, 2012)	*k* = −18→18
*T*_min_ = 0.685, *T*_max_ = 0.746	*l* = −20→20
63523 measured reflections	

### Refinement

**Table:**

Refinement on *F*^2^	0 restraints
Least-squares matrix: full	Hydrogen site location: mixed
*R*[*F*^2^ > 2σ(*F*^2^)] = 0.050	H-atom parameters constrained
*wR*(*F*^2^) = 0.146	*w* = 1/[σ^2^(*F*_o_^2^) + (0.0677*P*)^2^ + 3.3297*P*] where *P* = (*F*_o_^2^ + 2*F*_c_^2^)/3
*S* = 1.07	(Δ/σ)_max_ < 0.001
10700 reflections	Δρ_max_ = 0.57 e Å^−^^3^
602 parameters	Δρ_min_ = −1.75 e Å^−^^3^

### Special details

**Table:**

Experimental. Absorption correction: SADABS-2012/1 (Bruker,2012) was used for absorption correction. wR2(int) was 0.0499 before and 0.0451 after correction. The Ratio of minimum to maximum transmission is 0.9191. The λ/2 correction factor is 0.0015.
Geometry. All e.s.d.'s (except the e.s.d. in the dihedral angle between two l.s. planes) are estimated using the full covariance matrix. The cell e.s.d.'s are taken into account individually in the estimation of e.s.d.'s in distances, angles and torsion angles; correlations between e.s.d.'s in cell parameters are only used when they are defined by crystal symmetry. An approximate (isotropic) treatment of cell e.s.d.'s is used for estimating e.s.d.'s involving l.s. planes.

### Fractional atomic coordinates and isotropic or equivalent isotropic displacement parameters (Å^2^)

**Table:**

	*x*	*y*	*z*	*U*_iso_*/*U*_eq_	
C1	1.20881 (18)	0.42471 (15)	0.59436 (13)	0.0125 (4)	
C2	1.2657 (2)	0.46241 (16)	0.64338 (14)	0.0155 (4)	
H2	1.3348	0.4212	0.6669	0.019*	
C3	1.2026 (2)	0.56745 (16)	0.64990 (14)	0.0159 (4)	
H3	1.2186	0.6136	0.6796	0.019*	
C4	1.10711 (19)	0.59648 (15)	0.60380 (13)	0.0124 (4)	
C5	1.02670 (19)	0.69995 (15)	0.59077 (13)	0.0128 (4)	
C6	0.93703 (19)	0.72775 (15)	0.54243 (13)	0.0126 (4)	
C7	0.8508 (2)	0.83289 (16)	0.53189 (14)	0.0154 (4)	
H7	0.8481	0.8947	0.5527	0.018*	
C8	0.7741 (2)	0.82731 (16)	0.48687 (14)	0.0160 (4)	
H8	0.7079	0.8845	0.4699	0.019*	
C9	0.81131 (19)	0.71873 (15)	0.46954 (13)	0.0127 (4)	
C10	0.75323 (19)	0.68074 (15)	0.42513 (13)	0.0127 (4)	
C11	0.63875 (19)	0.75727 (15)	0.39930 (14)	0.0136 (4)	
C12	0.6415 (2)	0.78155 (17)	0.31039 (15)	0.0179 (4)	
H12	0.7195	0.7525	0.2645	0.021*	
C13	0.5315 (2)	0.84799 (17)	0.28738 (15)	0.0202 (4)	
H13	0.5348	0.8646	0.2263	0.024*	
C14	0.4173 (2)	0.88964 (16)	0.35441 (16)	0.0201 (4)	
C15	0.4140 (2)	0.86738 (18)	0.44410 (16)	0.0230 (5)	
H15	0.3362	0.8969	0.4902	0.028*	
C16	0.5238 (2)	0.80237 (17)	0.46579 (15)	0.0184 (4)	
H16	0.5211	0.7881	0.5271	0.022*	
C17	0.3015 (3)	0.9781 (2)	0.24935 (19)	0.0339 (6)	
H17A	0.3293	0.9142	0.2145	0.051*	
H17B	0.3591	1.0120	0.2231	0.051*	
H17C	0.2152	1.0255	0.2482	0.051*	
C18	1.03715 (19)	0.78442 (15)	0.63316 (14)	0.0139 (4)	
C19	1.0706 (2)	0.86013 (16)	0.58204 (15)	0.0170 (4)	
H19	1.0844	0.8597	0.5188	0.020*	
C20	1.0840 (2)	0.93562 (17)	0.62209 (15)	0.0194 (4)	
H20	1.1058	0.9871	0.5865	0.023*	
C21	1.0655 (2)	0.93647 (16)	0.71476 (16)	0.0191 (4)	
C22	1.0296 (2)	0.86335 (17)	0.76751 (15)	0.0178 (4)	
H22	1.0150	0.8645	0.8308	0.021*	
C23	1.0154 (2)	0.78829 (16)	0.72589 (14)	0.0158 (4)	
H23	0.9903	0.7385	0.7618	0.019*	
C24	1.0669 (3)	1.0151 (2)	0.8425 (2)	0.0389 (7)	
H24A	1.0857	1.0712	0.8573	0.058*	
H24B	1.1243	0.9482	0.8602	0.058*	
H24C	0.9788	1.0278	0.8748	0.058*	
N1	1.11191 (16)	0.50834 (13)	0.57123 (11)	0.0115 (3)	
N2	1.08769 (16)	0.34051 (13)	0.49707 (11)	0.0117 (3)	
O1	1.08434 (18)	1.01222 (13)	0.74747 (12)	0.0272 (4)	
O2	0.30280 (16)	0.95252 (14)	0.34014 (12)	0.0308 (4)	
O3	1.13810 (14)	0.49687 (11)	0.37896 (10)	0.0154 (3)	
H3A	1.1366	0.5587	0.3672	0.023*	
H3B	1.1244	0.4756	0.3342	0.023*	
Fe1	1.0000	0.5000	0.5000	0.01050 (10)	
C32	0.5833 (2)	0.3301 (2)	0.33522 (18)	0.0273 (5)	
H32A	0.6263	0.3492	0.2759	0.033*	
H32B	0.6491	0.2844	0.3644	0.033*	
C33	0.4953 (2)	0.4268 (2)	0.39253 (17)	0.0268 (5)	
H33A	0.4302	0.4120	0.4404	0.032*	
H33B	0.5439	0.4477	0.4217	0.032*	
C34	0.3596 (2)	0.6035 (2)	0.39343 (18)	0.0280 (5)	
H34A	0.4150	0.6242	0.4154	0.034*	
H34B	0.2979	0.5914	0.4463	0.034*	
C35	0.2903 (2)	0.6890 (2)	0.33939 (18)	0.0278 (5)	
H35A	0.2540	0.7568	0.3719	0.033*	
H35B	0.3498	0.6923	0.2809	0.033*	
C36	0.1311 (3)	0.7425 (2)	0.26473 (19)	0.0321 (6)	
H36A	0.1951	0.7427	0.2078	0.038*	
H36B	0.0871	0.8137	0.2921	0.038*	
C37	0.0371 (2)	0.7098 (2)	0.24680 (19)	0.0304 (6)	
H37A	−0.0241	0.7059	0.3041	0.037*	
H37B	−0.0107	0.7613	0.2087	0.037*	
C38	0.0238 (2)	0.5666 (2)	0.19171 (17)	0.0260 (5)	
H38A	−0.0228	0.6071	0.1483	0.031*	
H38B	−0.0390	0.5687	0.2501	0.031*	
C39	0.1033 (3)	0.4563 (2)	0.15811 (17)	0.0275 (5)	
H39A	0.0506	0.4272	0.1421	0.033*	
H39B	0.1725	0.4536	0.1036	0.033*	
C40	0.2700 (2)	0.3065 (2)	0.18906 (17)	0.0282 (5)	
H40A	0.3348	0.3289	0.1496	0.034*	
H40B	0.2526	0.2643	0.1523	0.034*	
C41	0.3176 (2)	0.2432 (2)	0.26226 (18)	0.0318 (6)	
H41A	0.3257	0.2870	0.3036	0.038*	
H41B	0.2587	0.2124	0.2971	0.038*	
C42	0.5169 (2)	0.1182 (2)	0.27862 (18)	0.0294 (5)	
H42A	0.5746	0.0451	0.2583	0.035*	
H42B	0.4631	0.1169	0.3403	0.035*	
C43	0.5944 (2)	0.1764 (2)	0.28063 (18)	0.0291 (5)	
H43A	0.6574	0.1363	0.3137	0.035*	
H43B	0.6409	0.1852	0.2185	0.035*	
O7	0.19160 (16)	0.66968 (13)	0.32484 (12)	0.0257 (4)	
O8	0.10530 (16)	0.61021 (13)	0.20216 (12)	0.0255 (4)	
O9	0.15575 (16)	0.39614 (13)	0.22710 (11)	0.0232 (3)	
O10	0.43816 (17)	0.16351 (15)	0.22068 (12)	0.0314 (4)	
O11	0.51346 (16)	0.27633 (13)	0.32361 (11)	0.0244 (4)	
O12	0.43455 (16)	0.51013 (13)	0.33994 (12)	0.0254 (4)	
C26	0.7238 (3)	0.4969 (3)	0.1142 (2)	0.0439 (7)	
H26A	0.6682	0.4911	0.1738	0.053*	
H26B	0.7845	0.5216	0.1236	0.053*	
C27	0.5816 (3)	0.6691 (3)	0.1040 (2)	0.0412 (7)	
H27A	0.6420	0.6962	0.1101	0.049*	
H27B	0.5284	0.6645	0.1649	0.049*	
C28	0.5003 (3)	0.7409 (2)	0.0500 (2)	0.0390 (7)	
H28A	0.4569	0.8119	0.0778	0.047*	
H28B	0.5535	0.7443	−0.0114	0.047*	
C29	0.3298 (3)	0.7757 (3)	−0.0060 (2)	0.0472 (8)	
H29A	0.3819	0.7793	−0.0677	0.057*	
H29B	0.2867	0.8465	0.0221	0.057*	
C30	0.2338 (3)	0.7386 (2)	−0.0098 (2)	0.0452 (8)	
H30A	0.1869	0.7288	0.0519	0.054*	
H30B	0.1721	0.7911	−0.0389	0.054*	
C31	0.2060 (3)	0.6066 (3)	−0.0667 (2)	0.0431 (7)	
H31A	0.1494	0.6566	−0.1006	0.052*	
H31B	0.1531	0.6004	−0.0061	0.052*	
O13	0.64972 (18)	0.56855 (16)	0.06167 (12)	0.0327 (4)	
O14	0.4091 (2)	0.70619 (16)	0.04488 (14)	0.0374 (5)	
O15	0.29575 (19)	0.64266 (17)	−0.05957 (14)	0.0385 (5)	
O16	0.38103 (19)	0.49681 (16)	0.15978 (16)	0.0418 (5)	
H16A	0.3025	0.5226	0.1938	0.063*	
H16B	0.4320	0.4940	0.1904	0.063*	
K1	0.5000	0.5000	0.0000	0.0464 (2)	
C25	0.2987 (3)	0.0736 (2)	0.9826 (2)	0.0363 (6)	
O4	0.1767 (2)	0.2174 (2)	0.89228 (15)	0.0566 (7)	
O5	0.0631 (2)	0.1369 (2)	1.00466 (16)	0.0538 (6)	
O6	0.1311 (3)	0.25214 (18)	1.05230 (17)	0.0550 (7)	
F1	0.3295 (2)	0.00132 (16)	0.92180 (14)	0.0645 (6)	
F2	0.3945 (2)	0.1004 (2)	0.9639 (2)	0.0909 (10)	
F3	0.2917 (2)	0.02755 (16)	1.06154 (14)	0.0636 (6)	
S1	0.15173 (6)	0.18346 (5)	0.98227 (4)	0.02864 (15)	

### Atomic displacement parameters (Å^2^)

**Table:**

	*U*^11^	*U*^22^	*U*^33^	*U*^12^	*U*^13^	*U*^23^
C1	0.0104 (9)	0.0131 (9)	0.0134 (9)	−0.0034 (7)	−0.0047 (7)	0.0003 (7)
C2	0.0136 (9)	0.0157 (10)	0.0185 (10)	−0.0045 (8)	−0.0082 (8)	−0.0007 (8)
C3	0.0142 (9)	0.0154 (10)	0.0200 (10)	−0.0050 (8)	−0.0085 (8)	−0.0016 (8)
C4	0.0122 (9)	0.0140 (9)	0.0119 (9)	−0.0060 (8)	−0.0035 (7)	−0.0007 (7)
C5	0.0145 (9)	0.0118 (9)	0.0128 (9)	−0.0062 (8)	−0.0034 (7)	−0.0004 (7)
C6	0.0134 (9)	0.0105 (9)	0.0121 (9)	−0.0037 (7)	−0.0029 (7)	0.0001 (7)
C7	0.0171 (10)	0.0101 (9)	0.0188 (10)	−0.0041 (8)	−0.0071 (8)	0.0001 (7)
C8	0.0168 (10)	0.0113 (9)	0.0185 (10)	−0.0033 (8)	−0.0069 (8)	0.0006 (7)
C9	0.0122 (9)	0.0103 (9)	0.0127 (9)	−0.0019 (7)	−0.0036 (7)	−0.0001 (7)
C10	0.0103 (9)	0.0134 (9)	0.0130 (9)	−0.0027 (7)	−0.0047 (7)	0.0009 (7)
C11	0.0130 (9)	0.0100 (9)	0.0180 (10)	−0.0026 (7)	−0.0075 (8)	−0.0001 (7)
C12	0.0134 (10)	0.0184 (10)	0.0170 (10)	−0.0013 (8)	−0.0052 (8)	−0.0005 (8)
C13	0.0219 (11)	0.0173 (10)	0.0188 (10)	−0.0018 (9)	−0.0110 (9)	−0.0006 (8)
C14	0.0165 (10)	0.0127 (10)	0.0274 (12)	0.0026 (8)	−0.0127 (9)	−0.0038 (8)
C15	0.0146 (10)	0.0238 (11)	0.0224 (11)	0.0000 (9)	−0.0037 (9)	−0.0055 (9)
C16	0.0175 (10)	0.0177 (10)	0.0160 (10)	−0.0026 (8)	−0.0057 (8)	−0.0007 (8)
C17	0.0310 (14)	0.0269 (13)	0.0375 (15)	0.0054 (11)	−0.0257 (12)	−0.0027 (11)
C18	0.0109 (9)	0.0117 (9)	0.0183 (10)	−0.0025 (7)	−0.0057 (8)	−0.0016 (7)
C19	0.0165 (10)	0.0161 (10)	0.0171 (10)	−0.0057 (8)	−0.0045 (8)	0.0001 (8)
C20	0.0192 (10)	0.0152 (10)	0.0252 (11)	−0.0082 (8)	−0.0069 (9)	0.0014 (8)
C21	0.0178 (10)	0.0121 (9)	0.0282 (12)	−0.0021 (8)	−0.0120 (9)	−0.0045 (8)
C22	0.0183 (10)	0.0159 (10)	0.0176 (10)	−0.0027 (8)	−0.0082 (8)	−0.0031 (8)
C23	0.0157 (10)	0.0129 (9)	0.0170 (10)	−0.0036 (8)	−0.0054 (8)	0.0004 (7)
C24	0.0619 (19)	0.0242 (13)	0.0409 (16)	−0.0130 (13)	−0.0347 (15)	−0.0038 (11)
N1	0.0107 (8)	0.0098 (7)	0.0128 (8)	−0.0025 (6)	−0.0043 (6)	−0.0005 (6)
N2	0.0116 (8)	0.0110 (8)	0.0120 (8)	−0.0034 (6)	−0.0044 (6)	0.0006 (6)
O1	0.0375 (10)	0.0175 (8)	0.0359 (10)	−0.0113 (7)	−0.0220 (8)	−0.0024 (7)
O2	0.0201 (8)	0.0276 (9)	0.0339 (10)	0.0089 (7)	−0.0179 (7)	−0.0076 (7)
O3	0.0165 (7)	0.0148 (7)	0.0139 (7)	−0.0062 (6)	−0.0033 (6)	0.0001 (5)
Fe1	0.01064 (19)	0.00882 (19)	0.01182 (19)	−0.00256 (15)	−0.00491 (15)	−0.00027 (14)
C32	0.0217 (12)	0.0300 (13)	0.0318 (13)	−0.0119 (10)	−0.0087 (10)	0.0036 (10)
C33	0.0262 (12)	0.0302 (13)	0.0277 (12)	−0.0144 (10)	−0.0098 (10)	0.0041 (10)
C34	0.0227 (12)	0.0303 (13)	0.0296 (13)	−0.0110 (10)	−0.0032 (10)	−0.0057 (10)
C35	0.0237 (12)	0.0250 (12)	0.0344 (13)	−0.0127 (10)	−0.0028 (10)	−0.0019 (10)
C36	0.0352 (14)	0.0196 (12)	0.0389 (15)	−0.0095 (11)	−0.0124 (12)	0.0084 (10)
C37	0.0266 (13)	0.0219 (12)	0.0355 (14)	−0.0037 (10)	−0.0102 (11)	0.0067 (10)
C38	0.0233 (12)	0.0298 (13)	0.0248 (12)	−0.0095 (10)	−0.0105 (10)	0.0051 (10)
C39	0.0301 (13)	0.0314 (13)	0.0214 (11)	−0.0113 (11)	−0.0107 (10)	0.0026 (10)
C40	0.0273 (13)	0.0254 (12)	0.0234 (12)	−0.0035 (10)	−0.0038 (10)	−0.0072 (9)
C41	0.0241 (12)	0.0348 (14)	0.0246 (12)	−0.0003 (11)	−0.0058 (10)	−0.0064 (10)
C42	0.0259 (13)	0.0230 (12)	0.0327 (13)	−0.0013 (10)	−0.0109 (10)	−0.0043 (10)
C43	0.0215 (12)	0.0299 (13)	0.0273 (13)	−0.0030 (10)	−0.0052 (10)	−0.0035 (10)
O7	0.0238 (8)	0.0226 (8)	0.0301 (9)	−0.0106 (7)	−0.0074 (7)	0.0068 (7)
O8	0.0211 (8)	0.0247 (9)	0.0267 (9)	−0.0075 (7)	−0.0055 (7)	0.0040 (7)
O9	0.0223 (8)	0.0226 (8)	0.0189 (8)	−0.0041 (7)	−0.0050 (6)	−0.0010 (6)
O10	0.0217 (9)	0.0352 (10)	0.0270 (9)	0.0008 (8)	−0.0079 (7)	−0.0098 (8)
O11	0.0210 (8)	0.0259 (9)	0.0248 (8)	−0.0095 (7)	−0.0042 (7)	−0.0007 (7)
O12	0.0246 (9)	0.0244 (9)	0.0260 (9)	−0.0097 (7)	−0.0061 (7)	0.0007 (7)
C26	0.0436 (17)	0.066 (2)	0.0354 (15)	−0.0302 (16)	−0.0206 (13)	0.0076 (14)
C27	0.0373 (16)	0.0532 (18)	0.0305 (14)	−0.0210 (14)	0.0033 (12)	−0.0177 (13)
C28	0.0417 (16)	0.0365 (15)	0.0330 (15)	−0.0189 (13)	0.0053 (12)	−0.0104 (12)
C29	0.0525 (19)	0.0336 (16)	0.0535 (19)	−0.0162 (15)	−0.0198 (16)	0.0167 (14)
C30	0.0379 (16)	0.0346 (16)	0.0523 (19)	−0.0059 (13)	−0.0160 (14)	0.0147 (14)
C31	0.0319 (15)	0.0553 (19)	0.0440 (17)	−0.0172 (14)	−0.0187 (13)	0.0138 (14)
O13	0.0335 (10)	0.0413 (11)	0.0253 (9)	−0.0179 (9)	−0.0066 (8)	−0.0021 (8)
O14	0.0393 (11)	0.0363 (11)	0.0370 (11)	−0.0179 (9)	−0.0101 (9)	0.0066 (8)
O15	0.0311 (10)	0.0456 (12)	0.0395 (11)	−0.0164 (9)	−0.0126 (9)	0.0090 (9)
O16	0.0235 (10)	0.0375 (11)	0.0629 (14)	−0.0106 (9)	−0.0127 (9)	0.0002 (10)
K1	0.0438 (5)	0.0525 (6)	0.0406 (5)	−0.0206 (5)	−0.0075 (4)	0.0003 (4)
C25	0.0344 (15)	0.0311 (14)	0.0418 (16)	−0.0133 (12)	−0.0127 (12)	0.0118 (12)
O4	0.0476 (14)	0.0621 (15)	0.0347 (12)	−0.0033 (12)	−0.0124 (10)	0.0224 (11)
O5	0.0368 (12)	0.0881 (19)	0.0431 (13)	−0.0349 (13)	−0.0009 (10)	−0.0176 (12)
O6	0.0767 (18)	0.0347 (12)	0.0575 (15)	−0.0124 (12)	−0.0361 (13)	−0.0112 (10)
F1	0.0709 (15)	0.0407 (11)	0.0472 (12)	−0.0009 (10)	0.0011 (10)	−0.0089 (9)
F2	0.0396 (12)	0.0733 (16)	0.173 (3)	−0.0298 (12)	−0.0521 (16)	0.0453 (18)
F3	0.0876 (16)	0.0455 (11)	0.0465 (11)	−0.0116 (11)	−0.0333 (11)	0.0187 (9)
S1	0.0270 (3)	0.0356 (3)	0.0222 (3)	−0.0087 (3)	−0.0109 (2)	−0.0017 (2)

### Geometric parameters (Å, º)

**Table:**

C1—N1	1.378 (2)	C35—O7	1.419 (3)
C1—C10^i^	1.400 (3)	C35—H35A	0.9900
C1—C2	1.434 (3)	C35—H35B	0.9900
C2—C3	1.352 (3)	C36—O7	1.432 (3)
C2—H2	0.9500	C36—C37	1.502 (4)
C3—C4	1.436 (3)	C36—H36A	0.9900
C3—H3	0.9500	C36—H36B	0.9900
C4—N1	1.368 (3)	C37—O8	1.423 (3)
C4—C5	1.409 (3)	C37—H37A	0.9900
C5—C6	1.405 (3)	C37—H37B	0.9900
C5—C18	1.492 (3)	C38—O8	1.422 (3)
C6—N2^i^	1.372 (3)	C38—C39	1.494 (4)
C6—C7	1.439 (3)	C38—H38A	0.9900
C7—C8	1.352 (3)	C38—H38B	0.9900
C7—H7	0.9500	C39—O9	1.431 (3)
C8—C9	1.437 (3)	C39—H39A	0.9900
C8—H8	0.9500	C39—H39B	0.9900
C9—N2^i^	1.377 (2)	C40—O9	1.433 (3)
C9—C10	1.399 (3)	C40—C41	1.478 (4)
C10—C1^i^	1.400 (3)	C40—H40A	0.9900
C10—C11	1.494 (3)	C40—H40B	0.9900
C11—C12	1.388 (3)	C41—O10	1.419 (3)
C11—C16	1.393 (3)	C41—H41A	0.9900
C12—C13	1.395 (3)	C41—H41B	0.9900
C12—H12	0.9500	C42—O10	1.421 (3)
C13—C14	1.388 (3)	C42—C43	1.499 (4)
C13—H13	0.9500	C42—H42A	0.9900
C14—O2	1.366 (3)	C42—H42B	0.9900
C14—C15	1.394 (3)	C43—O11	1.427 (3)
C15—C16	1.379 (3)	C43—H43A	0.9900
C15—H15	0.9500	C43—H43B	0.9900
C16—H16	0.9500	C26—O13	1.416 (4)
C17—O2	1.428 (3)	C26—C31^ii^	1.486 (5)
C17—H17A	0.9800	C26—H26A	0.9900
C17—H17B	0.9800	C26—H26B	0.9900
C17—H17C	0.9800	C27—O13	1.419 (4)
C18—C23	1.393 (3)	C27—C28	1.479 (5)
C18—C19	1.396 (3)	C27—H27A	0.9900
C19—C20	1.380 (3)	C27—H27B	0.9900
C19—H19	0.9500	C28—O14	1.413 (4)
C20—C21	1.395 (3)	C28—H28A	0.9900
C20—H20	0.9500	C28—H28B	0.9900
C21—O1	1.362 (3)	C29—O14	1.420 (4)
C21—C22	1.389 (3)	C29—C30	1.484 (5)
C22—C23	1.395 (3)	C29—H29A	0.9900
C22—H22	0.9500	C29—H29B	0.9900
C23—H23	0.9500	C30—O15	1.419 (4)
C24—O1	1.434 (3)	C30—H30A	0.9900
C24—H24A	0.9800	C30—H30B	0.9900
C24—H24B	0.9800	C31—O15	1.420 (4)
C24—H24C	0.9800	C31—C26^ii^	1.486 (5)
N1—Fe1	2.0388 (16)	C31—H31A	0.9900
N2—C6^i^	1.372 (3)	C31—H31B	0.9900
N2—C9^i^	1.377 (2)	O13—K1	2.8108 (19)
N2—Fe1	2.0468 (16)	O14—K1	2.731 (2)
O3—Fe1	2.1048 (14)	O15—K1	2.798 (2)
O3—H3A	0.8699	O16—K1	2.484 (2)
O3—H3B	0.8715	O16—H16A	0.8742
Fe1—N1^i^	2.0387 (16)	O16—H16B	0.8734
Fe1—N2^i^	2.0469 (16)	K1—O16^ii^	2.484 (2)
Fe1—O3^i^	2.1049 (14)	K1—O14^ii^	2.731 (2)
C32—O11	1.414 (3)	K1—O15^ii^	2.798 (2)
C32—C33	1.501 (4)	K1—O13^ii^	2.8108 (19)
C32—H32A	0.9900	K1—H16B	2.8356
C32—H32B	0.9900	C25—F2	1.314 (4)
C33—O12	1.426 (3)	C25—F1	1.323 (4)
C33—H33A	0.9900	C25—F3	1.329 (3)
C33—H33B	0.9900	C25—S1	1.803 (3)
C34—O12	1.423 (3)	O4—S1	1.422 (2)
C34—C35	1.495 (4)	O5—S1	1.429 (2)
C34—H34A	0.9900	O6—S1	1.432 (2)
C34—H34B	0.9900		
			
N1—C1—C10^i^	126.52 (18)	H37A—C37—H37B	108.4
N1—C1—C2	109.00 (17)	O8—C38—C39	108.3 (2)
C10^i^—C1—C2	124.47 (18)	O8—C38—H38A	110.0
C3—C2—C1	107.28 (18)	C39—C38—H38A	110.0
C3—C2—H2	126.4	O8—C38—H38B	110.0
C1—C2—H2	126.4	C39—C38—H38B	110.0
C2—C3—C4	107.47 (18)	H38A—C38—H38B	108.4
C2—C3—H3	126.3	O9—C39—C38	108.9 (2)
C4—C3—H3	126.3	O9—C39—H39A	109.9
N1—C4—C5	126.13 (18)	C38—C39—H39A	109.9
N1—C4—C3	109.11 (17)	O9—C39—H39B	109.9
C5—C4—C3	124.69 (18)	C38—C39—H39B	109.9
C6—C5—C4	124.34 (18)	H39A—C39—H39B	108.3
C6—C5—C18	118.69 (17)	O9—C40—C41	109.3 (2)
C4—C5—C18	116.97 (18)	O9—C40—H40A	109.8
N2^i^—C6—C5	125.68 (18)	C41—C40—H40A	109.8
N2^i^—C6—C7	109.38 (17)	O9—C40—H40B	109.8
C5—C6—C7	124.83 (18)	C41—C40—H40B	109.8
C8—C7—C6	107.28 (18)	H40A—C40—H40B	108.3
C8—C7—H7	126.4	O10—C41—C40	106.6 (2)
C6—C7—H7	126.4	O10—C41—H41A	110.4
C7—C8—C9	107.27 (18)	C40—C41—H41A	110.4
C7—C8—H8	126.4	O10—C41—H41B	110.4
C9—C8—H8	126.4	C40—C41—H41B	110.4
N2^i^—C9—C10	126.08 (18)	H41A—C41—H41B	108.6
N2^i^—C9—C8	109.32 (17)	O10—C42—C43	112.4 (2)
C10—C9—C8	124.60 (18)	O10—C42—H42A	109.1
C9—C10—C1^i^	124.99 (18)	C43—C42—H42A	109.1
C9—C10—C11	118.12 (18)	O10—C42—H42B	109.1
C1^i^—C10—C11	116.85 (17)	C43—C42—H42B	109.1
C12—C11—C16	118.53 (19)	H42A—C42—H42B	107.9
C12—C11—C10	122.08 (18)	O11—C43—C42	109.9 (2)
C16—C11—C10	119.32 (18)	O11—C43—H43A	109.7
C11—C12—C13	121.1 (2)	C42—C43—H43A	109.7
C11—C12—H12	119.5	O11—C43—H43B	109.7
C13—C12—H12	119.5	C42—C43—H43B	109.7
C14—C13—C12	119.5 (2)	H43A—C43—H43B	108.2
C14—C13—H13	120.3	C35—O7—C36	111.93 (19)
C12—C13—H13	120.3	C38—O8—C37	112.56 (19)
O2—C14—C13	124.9 (2)	C39—O9—C40	110.73 (18)
O2—C14—C15	115.2 (2)	C41—O10—C42	114.24 (19)
C13—C14—C15	119.9 (2)	C32—O11—C43	111.96 (19)
C16—C15—C14	119.9 (2)	C34—O12—C33	110.40 (19)
C16—C15—H15	120.1	O13—C26—C31^ii^	108.9 (2)
C14—C15—H15	120.1	O13—C26—H26A	109.9
C15—C16—C11	121.1 (2)	C31^ii^—C26—H26A	109.9
C15—C16—H16	119.4	O13—C26—H26B	109.9
C11—C16—H16	119.4	C31^ii^—C26—H26B	109.9
O2—C17—H17A	109.5	H26A—C26—H26B	108.3
O2—C17—H17B	109.5	O13—C27—C28	109.9 (2)
H17A—C17—H17B	109.5	O13—C27—H27A	109.7
O2—C17—H17C	109.5	C28—C27—H27A	109.7
H17A—C17—H17C	109.5	O13—C27—H27B	109.7
H17B—C17—H17C	109.5	C28—C27—H27B	109.7
C23—C18—C19	118.14 (19)	H27A—C27—H27B	108.2
C23—C18—C5	120.10 (18)	O14—C28—C27	110.3 (2)
C19—C18—C5	121.75 (19)	O14—C28—H28A	109.6
C20—C19—C18	120.9 (2)	C27—C28—H28A	109.6
C20—C19—H19	119.5	O14—C28—H28B	109.6
C18—C19—H19	119.5	C27—C28—H28B	109.6
C19—C20—C21	120.1 (2)	H28A—C28—H28B	108.1
C19—C20—H20	119.9	O14—C29—C30	109.6 (2)
C21—C20—H20	119.9	O14—C29—H29A	109.7
O1—C21—C22	124.2 (2)	C30—C29—H29A	109.7
O1—C21—C20	115.7 (2)	O14—C29—H29B	109.7
C22—C21—C20	120.1 (2)	C30—C29—H29B	109.7
C21—C22—C23	118.8 (2)	H29A—C29—H29B	108.2
C21—C22—H22	120.6	O15—C30—C29	109.3 (3)
C23—C22—H22	120.6	O15—C30—H30A	109.8
C18—C23—C22	121.8 (2)	C29—C30—H30A	109.8
C18—C23—H23	119.1	O15—C30—H30B	109.8
C22—C23—H23	119.1	C29—C30—H30B	109.8
O1—C24—H24A	109.5	H30A—C30—H30B	108.3
O1—C24—H24B	109.5	O15—C31—C26^ii^	108.4 (3)
H24A—C24—H24B	109.5	O15—C31—H31A	110.0
O1—C24—H24C	109.5	C26^ii^—C31—H31A	110.0
H24A—C24—H24C	109.5	O15—C31—H31B	110.0
H24B—C24—H24C	109.5	C26^ii^—C31—H31B	110.0
C4—N1—C1	107.13 (16)	H31A—C31—H31B	108.4
C4—N1—Fe1	127.17 (13)	C26—O13—C27	111.1 (2)
C1—N1—Fe1	125.70 (13)	C26—O13—K1	115.41 (17)
C6^i^—N2—C9^i^	106.74 (16)	C27—O13—K1	113.69 (16)
C6^i^—N2—Fe1	127.25 (13)	C28—O14—C29	110.4 (2)
C9^i^—N2—Fe1	125.93 (13)	C28—O14—K1	115.77 (17)
C21—O1—C24	117.2 (2)	C29—O14—K1	114.69 (19)
C14—O2—C17	117.2 (2)	C30—O15—C31	110.7 (2)
Fe1—O3—H3A	110.7	C30—O15—K1	111.85 (17)
Fe1—O3—H3B	110.7	C31—O15—K1	116.69 (17)
H3A—O3—H3B	108.2	K1—O16—H16A	138.5
N1^i^—Fe1—N1	180.00 (6)	K1—O16—H16B	104.8
N1^i^—Fe1—N2	89.38 (7)	H16A—O16—H16B	109.9
N1—Fe1—N2	90.62 (7)	O16^ii^—K1—O16	180.0
N1^i^—Fe1—N2^i^	90.63 (7)	O16^ii^—K1—O14^ii^	81.03 (6)
N1—Fe1—N2^i^	89.37 (7)	O16—K1—O14^ii^	98.97 (6)
N2—Fe1—N2^i^	180.0	O16^ii^—K1—O14	98.97 (6)
N1^i^—Fe1—O3	89.90 (6)	O16—K1—O14	81.03 (6)
N1—Fe1—O3	90.10 (6)	O14^ii^—K1—O14	180.0
N2—Fe1—O3	87.90 (6)	O16^ii^—K1—O15^ii^	97.81 (6)
N2^i^—Fe1—O3	92.10 (6)	O16—K1—O15^ii^	82.19 (6)
N1^i^—Fe1—O3^i^	90.10 (6)	O14^ii^—K1—O15^ii^	61.71 (6)
N1—Fe1—O3^i^	89.90 (6)	O14—K1—O15^ii^	118.29 (6)
N2—Fe1—O3^i^	92.10 (6)	O16^ii^—K1—O15	82.19 (6)
N2^i^—Fe1—O3^i^	87.90 (6)	O16—K1—O15	97.81 (6)
O3—Fe1—O3^i^	180.00 (8)	O14^ii^—K1—O15	118.29 (6)
O11—C32—C33	109.6 (2)	O14—K1—O15	61.71 (6)
O11—C32—H32A	109.8	O15^ii^—K1—O15	180.0
C33—C32—H32A	109.8	O16^ii^—K1—O13	92.35 (6)
O11—C32—H32B	109.8	O16—K1—O13	87.64 (6)
C33—C32—H32B	109.8	O14^ii^—K1—O13	119.02 (6)
H32A—C32—H32B	108.2	O14—K1—O13	60.98 (6)
O12—C33—C32	111.0 (2)	O15^ii^—K1—O13	59.38 (6)
O12—C33—H33A	109.4	O15—K1—O13	120.62 (6)
C32—C33—H33A	109.4	O16^ii^—K1—O13^ii^	87.64 (6)
O12—C33—H33B	109.4	O16—K1—O13^ii^	92.36 (6)
C32—C33—H33B	109.4	O14^ii^—K1—O13^ii^	60.98 (6)
H33A—C33—H33B	108.0	O14—K1—O13^ii^	119.02 (6)
O12—C34—C35	110.2 (2)	O15^ii^—K1—O13^ii^	120.62 (6)
O12—C34—H34A	109.6	O15—K1—O13^ii^	59.37 (6)
C35—C34—H34A	109.6	O13—K1—O13^ii^	180.0
O12—C34—H34B	109.6	O16^ii^—K1—H16B	162.7
C35—C34—H34B	109.6	O16—K1—H16B	17.3
H34A—C34—H34B	108.1	O14^ii^—K1—H16B	101.1
O7—C35—C34	109.4 (2)	O14—K1—H16B	78.9
O7—C35—H35A	109.8	O15^ii^—K1—H16B	68.9
C34—C35—H35A	109.8	O15—K1—H16B	111.1
O7—C35—H35B	109.8	O13—K1—H16B	71.5
C34—C35—H35B	109.8	O13^ii^—K1—H16B	108.5
H35A—C35—H35B	108.2	F2—C25—F1	106.4 (3)
O7—C36—C37	108.0 (2)	F2—C25—F3	107.9 (3)
O7—C36—H36A	110.1	F1—C25—F3	106.6 (2)
C37—C36—H36A	110.1	F2—C25—S1	112.2 (2)
O7—C36—H36B	110.1	F1—C25—S1	111.6 (2)
C37—C36—H36B	110.1	F3—C25—S1	111.9 (2)
H36A—C36—H36B	108.4	O4—S1—O5	114.42 (16)
O8—C37—C36	108.2 (2)	O4—S1—O6	117.80 (17)
O8—C37—H37A	110.1	O5—S1—O6	112.46 (16)
C36—C37—H37A	110.1	O4—S1—C25	102.95 (14)
O8—C37—H37B	110.1	O5—S1—C25	102.34 (15)
C36—C37—H37B	110.1	O6—S1—C25	104.46 (15)
			
N1—C1—C2—C3	0.4 (2)	C5—C4—N1—Fe1	−3.1 (3)
C10^i^—C1—C2—C3	−179.0 (2)	C3—C4—N1—Fe1	−179.94 (13)
C1—C2—C3—C4	−0.9 (2)	C10^i^—C1—N1—C4	179.67 (19)
C2—C3—C4—N1	1.1 (2)	C2—C1—N1—C4	0.3 (2)
C2—C3—C4—C5	−175.8 (2)	C10^i^—C1—N1—Fe1	−1.2 (3)
N1—C4—C5—C6	0.9 (3)	C2—C1—N1—Fe1	179.41 (13)
C3—C4—C5—C6	177.28 (19)	C22—C21—O1—C24	0.6 (3)
N1—C4—C5—C18	179.63 (18)	C20—C21—O1—C24	−179.6 (2)
C3—C4—C5—C18	−4.0 (3)	C13—C14—O2—C17	−0.8 (3)
C4—C5—C6—N2^i^	1.6 (3)	C15—C14—O2—C17	179.6 (2)
C18—C5—C6—N2^i^	−177.13 (18)	O11—C32—C33—O12	−82.9 (2)
C4—C5—C6—C7	177.24 (19)	O12—C34—C35—O7	72.0 (2)
C18—C5—C6—C7	−1.5 (3)	O7—C36—C37—O8	−63.5 (3)
N2^i^—C6—C7—C8	0.5 (2)	O8—C38—C39—O9	67.5 (2)
C5—C6—C7—C8	−175.75 (19)	O9—C40—C41—O10	−172.8 (2)
C6—C7—C8—C9	0.4 (2)	O10—C42—C43—O11	68.4 (3)
C7—C8—C9—N2^i^	−1.2 (2)	C34—C35—O7—C36	−173.3 (2)
C7—C8—C9—C10	179.16 (19)	C37—C36—O7—C35	174.8 (2)
N2^i^—C9—C10—C1^i^	−1.6 (3)	C39—C38—O8—C37	−171.00 (19)
C8—C9—C10—C1^i^	178.0 (2)	C36—C37—O8—C38	172.9 (2)
N2^i^—C9—C10—C11	175.89 (18)	C38—C39—O9—C40	−155.4 (2)
C8—C9—C10—C11	−4.5 (3)	C41—C40—O9—C39	−176.9 (2)
C9—C10—C11—C12	109.9 (2)	C40—C41—O10—C42	159.3 (2)
C1^i^—C10—C11—C12	−72.4 (3)	C43—C42—O10—C41	−87.5 (3)
C9—C10—C11—C16	−73.1 (3)	C33—C32—O11—C43	−171.6 (2)
C1^i^—C10—C11—C16	104.5 (2)	C42—C43—O11—C32	174.1 (2)
C16—C11—C12—C13	−1.2 (3)	C35—C34—O12—C33	−176.73 (19)
C10—C11—C12—C13	175.8 (2)	C32—C33—O12—C34	−175.17 (19)
C11—C12—C13—C14	−0.7 (3)	O13—C27—C28—O14	62.5 (3)
C12—C13—C14—O2	−177.6 (2)	O14—C29—C30—O15	−66.2 (3)
C12—C13—C14—C15	1.9 (3)	C31^ii^—C26—O13—C27	−179.4 (2)
O2—C14—C15—C16	178.3 (2)	C31^ii^—C26—O13—K1	49.3 (3)
C13—C14—C15—C16	−1.3 (4)	C28—C27—O13—C26	−177.2 (2)
C14—C15—C16—C11	−0.6 (4)	C28—C27—O13—K1	−45.0 (3)
C12—C11—C16—C15	1.9 (3)	C27—C28—O14—C29	179.3 (2)
C10—C11—C16—C15	−175.2 (2)	C27—C28—O14—K1	−48.3 (3)
C6—C5—C18—C23	119.8 (2)	C30—C29—O14—C28	−179.4 (3)
C4—C5—C18—C23	−59.0 (3)	C30—C29—O14—K1	47.6 (3)
C6—C5—C18—C19	−61.3 (3)	C29—C30—O15—C31	−178.3 (2)
C4—C5—C18—C19	119.9 (2)	C29—C30—O15—K1	49.8 (3)
C23—C18—C19—C20	1.1 (3)	C26^ii^—C31—O15—C30	−176.5 (2)
C5—C18—C19—C20	−177.80 (19)	C26^ii^—C31—O15—K1	−47.1 (3)
C18—C19—C20—C21	0.7 (3)	F2—C25—S1—O4	61.5 (3)
C19—C20—C21—O1	178.2 (2)	F1—C25—S1—O4	−57.7 (2)
C19—C20—C21—C22	−2.0 (3)	F3—C25—S1—O4	−177.0 (2)
O1—C21—C22—C23	−178.8 (2)	F2—C25—S1—O5	−179.5 (3)
C20—C21—C22—C23	1.4 (3)	F1—C25—S1—O5	61.2 (2)
C19—C18—C23—C22	−1.7 (3)	F3—C25—S1—O5	−58.1 (3)
C5—C18—C23—C22	177.21 (19)	F2—C25—S1—O6	−62.1 (3)
C21—C22—C23—C18	0.5 (3)	F1—C25—S1—O6	178.6 (2)
C5—C4—N1—C1	176.04 (19)	F3—C25—S1—O6	59.3 (3)
C3—C4—N1—C1	−0.8 (2)		

Symmetry codes: (i) −*x*+2, −*y*+1, −*z*+1; (ii) −*x*+1, −*y*+1, −*z*.

### Hydrogen-bond geometry (Å, º)

Cg1, Cg2, Cg3 and Cg9 ae the centroids of the N1/C1–C4, N2/C6'–C9', N2'/C6–C9 and C18–C23 rings, respectively.

**Table:**

*D*—H···*A*	*D*—H	H···*A*	*D*···*A*	*D*—H···*A*
O3—H3*B*···O9^iii^	0.87	1.96	2.797 (2)	160
O3—H3*A*···O7^iii^	0.87	1.94	2.778 (2)	161
O16—H16*A*···O8	0.87	2.15	2.922 (3)	147
O16—H16*B*···O12	0.87	2.38	3.107 (3)	141
C7—H7···O2^iv^	0.95	2.52	3.303 (3)	139
C12—H12···O4^v^	0.95	2.48	3.270 (3)	141
C16—H16···O11^v^	0.95	2.39	3.319 (3)	164
C22—H22···O5^vi^	0.95	2.46	3.390 (3)	168
C24—H24*A*···O5^vii^	0.98	2.46	3.184 (4)	130
C28—H28*A*···F2^v^	0.99	2.50	2.983 (4)	110
C30—H30*B*···O5^viii^	0.99	2.49	3.248 (5)	133
C38—H38*A*···O4^viii^	0.99	2.55	3.429 (4)	148
C40—H40*B*···O6^ix^	0.99	2.51	3.402 (4)	150
C41—H41*A*···O11	0.99	2.33	3.030 (4)	127
C17—H17*C*···*Cg*9^iv^	0.98	2.84	3.753 (4)	155
C34—H34*B*···*Cg*1^x^	0.99	2.93	3.894 (3)	164
C38—H38*B*···*Cg*1^v^	0.99	2.78	3.706 (3)	155
C41—H41*B*···*Cg*2^x^	0.99	2.95	3.584 (3)	123
C41—H41*B*···*Cg*3^v^	0.99	2.95	3.584 (3)	123

Symmetry codes: (iii) *x*+1, *y*, *z*; (iv) −*x*+1, −*y*+2, −*z*+1; (v) −*x*+1, −*y*+1, −*z*+1; (vi) −*x*+1, −*y*+1, −*z*+2; (vii) *x*+1, *y*+1, *z*; (viii) −*x*, −*y*+1, −*z*+1; (ix) *x*, *y*, *z*−1; (x) *x*−1, *y*, *z*.

## References

[bb2] Ben Haj Hassen, L., Ezzayani, K., Rousselin, Y. & Nasri, H. (2014). *Acta Cryst.* E**70**, m296–m297. 10.1107/S1600536814015335 PMC4158530 25249880

[bb3] Bourhis, L. J., Dolomanov, O. V., Gildea, R. J., Howard, J. A. K. & Puschmann, H. (2015). *Acta Cryst.* A**71**, 59–75. 10.1107/S2053273314022207 PMC4283469 25537389

[bb4] Bruker (2012). *SADABS*. Bruker AXS Inc., Madison, Wisconsin, USA.

[bb5] Bruker (2013). *APEX2* and *SAINT*. Bruker AXS Inc., Madison, Wisconsin, USA.

[bb6] Cheng, B., Safo, M. K., Orosz, R. D., Reed, C. A., Debrunner, P. G. & Scheidt, W. R. (1994). *Inorg. Chem.* **33**, 1319–1324.

[bb7] Denden, Z., Ezzayani, K., Saint-Aman, E., Loiseau, F., Najmudin, S., Bonifácio, C., Daran, J.-C. & Nasri, H. (2015). *Eur. J. Inorg. Chem.* pp. 2596–2610.

[bb8] Dolomanov, O. V., Bourhis, L. J., Gildea, R. J., Howard, J. A. K. & Puschmann, H. (2009). *J. Appl. Cryst.* **42**, 339–341.

[bb9] Gismelseed, A., Bominaar, E. L., Bill, E., Trautwein, A. X., Winkler, H., Nasri, H., Doppelt, P., Mandon, D., Fischer, J. & Weiss, R. (1990). *Inorg. Chem.* **29**, 2741–2749.

[bb10] Scheidt, W. R., Cohen, I. A. & Kastner, M. E. (1979). *Biochemistry*, **18**, 3546–3552. 10.1021/bi00583a017 224911

[bb11] Scheidt, W. R. & Reed, C. A. (1981). *J. Am. Chem. Soc.* **81**, 543–555.

[bb12] Sheldrick, G. M. (2008). *Acta Cryst.* A**64**, 112–122. 10.1107/S0108767307043930 18156677

[bb13] Xu, N., Powell, D. R. & Richter-Addo, G. B. (2011). *Angew. Chem. Int. Ed.* **50**, 9694–9696. 10.1002/anie.201103329 21913289

