# Supplementary figures and images for: Crystal structure of di­aqua­[5,10,15,20-tetra­kis­(4-meth­oxy­phen­yl)porphyrinato-κ4 N]iron(III) di­aqua­(18-crown-6)potassium bis­(tri­fluoro­methane­sulfonate)–18-crown-6 (1/2)
Source: Acta Crystallogr E Crystallogr Commun. 2015 Nov 11;71(Pt 12):m215–6. doi: 10.1107/S2056989015021039 (PMC4719840; doi:10.1107/S2056989015021039)

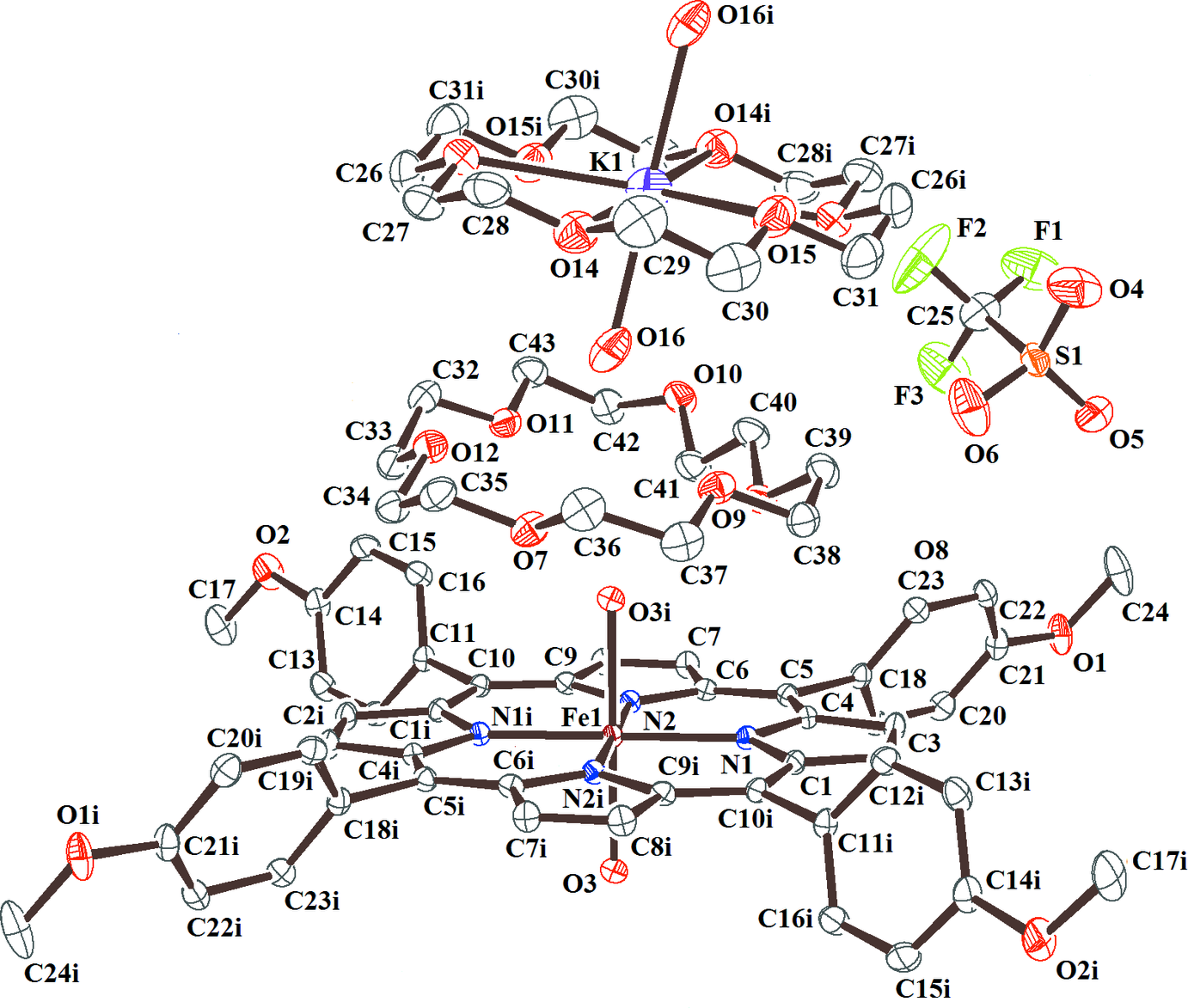

Supplement: Supplementary file 3 [file e-71-0m215-fig1.tif]

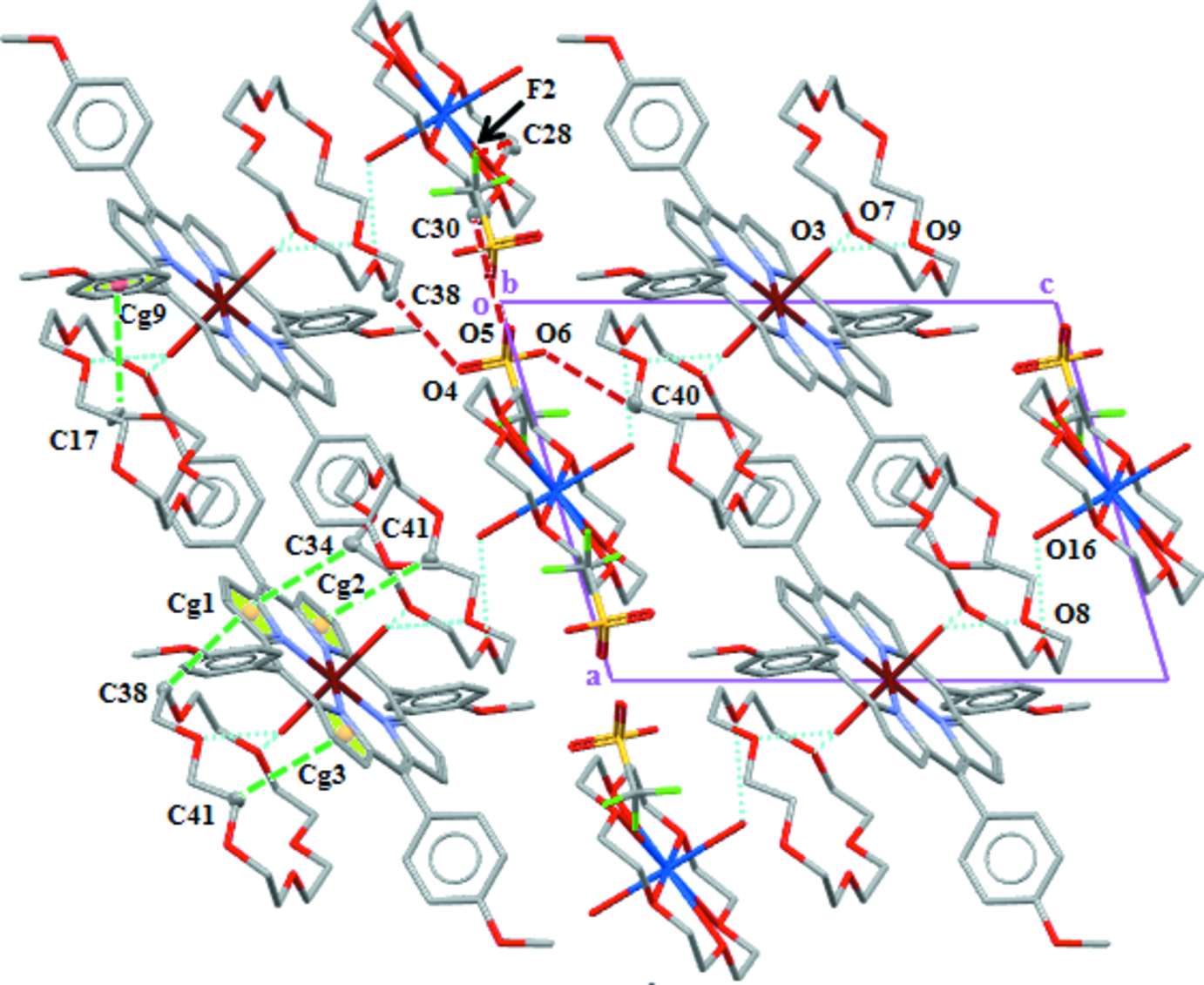

Supplement: Supplementary file 4 [file e-71-0m215-fig2.tif]
